# 

**Published:** 1865-08

**Authors:** 


### INDEX TO VOL. V.

ORIGINAL DEPARTMENT.

Action of Medicines on the Blood-vessels, - - - - - - - - - 4–41
Amputations, - - - - - - - - - - - - - - 18
Abstract of the proceedings of the Buffalo Medical Association, - 56, 95, 135, 202, 233, 271, 314, 384, 432, 476.
Arsenic, acute poisoning, by J. R. Lothrop, M. D., - - - - - - 66
Abstract of the proceedings of Medical Society of South-western New York, 140
Address, by Dr. Wm. Gould, - - - - - - - - - - - 433
Abscess, Lumbar, - - - - - - - - - - - - - - 238
Blood-vessels, Influence of heat on the caliber - - - - - - - 11
Blood-vessels, effect of medicinal substances on caliber, - - - - - 13
Buffalo General Hospital, Report of, by J. R. Lothrop, M. D., - - - 17
Blood-poison, - - - - - - - - - - - - - - - 221
Brain, gunshot wound of, - - - - - - - - - - - 223
Bellevue Medical College, Report of clinical cases, by Frank King, - - 279
Bladder, Extroversion of, - - - - - - - - - - - 280
Buffalo General Hospital, clinical cases in, by J. F. Miner, M.D., 137, 236, 275, 311
Cancer, - - - - - - - - - - - - - - - - - 1
Cancer, in Buffalo General Hospital, - - - - - - - - - 18
Cancer of the Glans Penis, - - - - - - - - - - - 86
Clinical remarks on Surgical cases in Buffalo General Hospital, by J. F. Miner, M. D., - - - - - - - 137, 236, 275, 311
Cholera, translated from the German, by T. A. McGraw, M. D., - - 181
Calculus, Phosphatic, - - - - - - - - - - - - - 221
Cranial bones, Syphilitic necrosis, by J. F. Miner, M. D., - - - - 311
Cholera, Action taken by physicians of Buffalo on, - - - - - - 388
Cholera, Contagious, by J. Biglow, M. D., - - - - - - - 319
Remarks on Cholera, by Thomas F. Rochester, M. D., - - - - - 461
Dysentery, - - - - - - - - - - - - - - - - 219
Disease of the Eye, - - - - - - - - - - - - - 19
Disease of the Foot, - - - - - - - - - - - - - 215
Disease of the Heart, - - - - - - - - - - - - - 216
Eye, Repeated evacuation of aqueous humor, - - - - - - - - 323
Eye, Diseases of, in Buffalo General Hospital, - - - - - - - 19
Erysipelatous pneumonia, - - - - - - - - - - - - 126
Exsection of the head of the humerus, by J. F. Miner, M. D., - - 139, 236
Excision of humerus, by J. F. Miner, M. D., - - - - - - - - 236
Excision of the hip joint, by J. F. Miner, M. D., - - - - - - - 276
Excision of the elbow in Bellevue Hospital College, - - - - - - 280
Extroversion of bladder, - - - - - - - - - - - - 280
Eclampsia, by M. W. Townsend, M. D., - - - - - - - - - 316
Eye, optical defects of, and their treatment by the use of spectacles, by A. M. Rosebrugh, M. D., - - - - - - - - - - 255, 326
Fractures in Buffalo General Hospital, - - - - - - - - 18
Febrile phenomena, explanation of, - - - - - - - - - 50
Febrile phenomena, increased action of Heart, - - - - - - - 52
Fistula, vesico-vaginal, by S. Barrett M. D., - - - - - - - - 89
Fibrous tumor of the uterus, - - - - - - - - - - - 220
Fungus Hæmatodes, - - - - - - - - - - - - - 279
Foreign substances simultaneously in the Trachea and Œsophagus, report of a case, by J. F. Miner, M. D., - - - - - - - - - - 471

Gunshot wounds in Buffalo General Hospital, 20
Glans Penis, cancer of, 86
Gunshot wound of the brain, 223
Gunshot wound, with removal of the acetabulum and dislocation of femur, by J. F. Miner, M. D., 381
Gunshot wound of Femur, by J. R. Lothrop, M. D., 465
Heart, atrophy of, 3
Heart, influence of, on the caliber of blood-vessels, 11
Humerus excision of head, by J. F. Miner, M. D., 139, 236
Heart, rupture of, 216
Heart, valvular disease of, 216
Hemiplegia of right side, 135
Hæmorrhage, Post Partum, by E. W. Jenks, M. D., 225
Hip Joint, excision of, by J. F. Miner, M. D., 275
Hæmatodes, fungus, 279
Hospital Cases, reported by Frank King, M. D., 440
Hydrophobia, report of case by Dr. Stiles, 87
Influence of Heat on caliber of vessels, 11
Impassible stricture, operation, by J. F. Miner M. D., 271
Intra-uterine tumor, removal of, 281
Infantile Mortality in New York city, by Cyrus Ramsy, M. D., LL. B., 475
Kings County Medical Society, transactions of, 1, 41, 85, 125, 171, 215
Lothrop, J. R., M. D., report of Buffalo General Hospital, 17
Lothrop, J. R., M. D., case of acute poisoning by arsenic, 66
Lothrop, J. R., M. D., case of gunshot wound of femur, 465
Lumbar abscess, case by J. F. Miner, M. D., 238, 275
Miner, J. F., M. D., orthopoedic surgery, 59, 91
" " " remarks on Talipes, 91
" " " removal bony tumor from shoulder joint 95
" " " clinical remarks on surgical cases in Buffalo General Hospital 137, 236, 275, 311
Miner, J. F., M. D., operation for impassible stricture, 271
" " " varicose veins, 137, 238
" " " excision of the head of humerus, 139
" " " two cases of excision of humerus, 236
" " " lumbar abscess, 238, 275
" " " excision of hip joint, 276
" " " fatty tumor, removal, 278
" " " syphilitic necrosis of skull, 311
" " " Trephining, 311
" " " gunshot wound, removal of acetabulum and dislocation of femur, recovery, 381
Miner, J. F., M. D., Ovariotomy, removal of ovarian tumor weighing twenty pounds, recovery, 421
Miner, J. F., M. D., Foreign Substances in Trachea and Œsophagus, 471
Military Surgery, 125
Necrosis of the Skull, 311
Orthopoetic Surgery, cases and remarks, 59, 91
Otitis, perforating mastoid portion of temporal bone with a trephine, by Dr. J. C. Hutchison, 85
Optical Defects of the Eye, by A. M. Rosebrugh, M. D., 255, 326
Opium Poisoning, case, by M. W. Townsend. 316
Ovariotomy, removal of ovarian tumor weighing twenty pounds, recovery, 421
Polypus, specimen, by Dr. Colgan, 2
Pyæmia, 3
Polypus of the Womb, case, by Dr. Hart, 4
Paralysis, cases in Buffalo General Hospital, 19
Poisoning by Arsenic, ending in recovery, by J. R. Lothrop, M. D., 66
Pneumonia Erysipelatous, by Dr. W. C. Otterson, 125
Phosphatic Calculus, by Dr. Hutchison, 221
Post Partum Hæmorrhage, by E. W. Jenks, M. D., 225

Report of the Buffalo General Hospital, by J. R. Lothrop, M. D., - - 17
Rupture of the Heart, by Dr. Speir. - - - - - - - - - - - 216
Rosebrugh, A. M., M. D., Optical Defects of the Eye, - - - - - 255, 325
Report of a case of the successful removal of a multilocular ovarian tumor weighing twenty pounds, - - - - - - - - - - - 421
Rochester, T. F., M. D., Remarks on Cholera, - - - - - - - - 461
Specimen, by Dr. S. F. Speir, - - - - - - - - - - - - 3
Small Pox in Cities, causes of its spreading and means for checking it, by J. B. Jones, M. D., - - - - - - - - - - - - - - 171
Specimens presented, by Dr. Stiles, - - - - - - - - - - - 215
Stricture, operation for impassible, - - - - - - - - - - - 271
Syphilitic Necrosis of the Skull, - - - - - - - - - - - - 311
Stone in the Male and Female, reported by Frank King, M. D., - - 441
Transactions of the Medical Society of County of Kings, - 1, 41, 85, 125. 171, 215
Testicle, Tuberculosis of, by Dr. Bartlett, - - - - - - - - - - 2
Talipes, Remarks on, - - - - - - - - - - - - - - - 91
Treatment of Wounds, by W. C. Otterson, M. D., - - - - - - 128
Tubercular Disease of the Foot, by Dr. Stiles, - - - - - - - - 215
Typhoid Fever, by Dr. Stiles, , - - - - - - - - - - - - 217
Tumor, Fatty, Removal of, - - - - - - - - - - - - - - 278
Trephining, - - - - - - - - - - - - - - - - - 311
Uterus, Fibrous Tumor of, by Dr. Hutchison, - - - - - - - - 220
Ulcers, case in Buffalo General Hospital, - - - - - - - - - - 236
Urinary Calculi, cases reported by Frank King, M. D., - - - - - 440
Venereal Disease. cases in Buffalo General Hospital, - - - - - - 20
Vesico-Vaginal Fistula, cases, by S. Barrett, M. D., - - - - - - 89
Varicose Veins, cases in Buffalo General Hospital, - - - - - 137, 236
Valvular Disease of the Heart, - - - - - - - - - - - - 216
Womb, Polypus of, by Dr. Hart, - - - - - - - - - - - 4
Wounds, Gun-shot, in Buffalo General Hospital, - - - - - - - - 20
Wounds, by W. C. Otterson, M. D., - - - - - - - - - - - 125
Wounds, Treatment of, - - - - - - - - - - - - - - 128

### CORRESPONDENCE.

Autopsy, report of a case, Ira D. Hopkins, M. D., - - - - - - - - 22
Cholera, by J. L. Page, M. D., - - - - - - - - - - - - 207
Cholera, by C. A. Lee, M. D., - - - - - - - - - - - - 292
Cutaneous Diseases cured without Mercury, by Wm. M. Cornell, M.D., LL.D., 406
Cure of Hydrocele, a case, by H. Rogers, M. D., - - - - - - - - 453
Dr. Kempson, Letter from, - - - - - - - - - - - - 412
Dr. Marsden's, Plan of Quarantine, - - - - - - - - - - 293
Military Surgery, by O. D. Norton, - - - - - - - - - - 206
Quarantine, - - - - - - - - - - - - - - - - - 293
Rochester, T. F., M. D., Letter from, - - - - - - - - - - 484
Suppression of Urine, by I. D. Hopkins, M. D., - - - - - - - 345
White, Prof. James P., Correspondeuce while on a tour in Europe, 341, 399, 450, 477.

### SUMMARY.

Amorphous Oxide of Mercury, yellow, - - - - - - - - - - 23
Cerebro-Spinal Meningitis, by S. Wilks, M. D., - - - - - - - - 25
Circulation, A New, by D. B. Jones, M. D., - - - - - - - - - 77
Diphtheria, Lecture on, by E. H. Greenhow, M. D., - - - - - - - 100
Epidemic at Maplewood Ladies' Institute, - - - - - - - - - 202
Fibroid Tumor, Recurrent, by F. H. Morris, M. D., - - - - - - - 26
Fever, Treatment of, by Dr. Gull, - - - - - - - - - - - 74
Hydrocele, Treatment of, by J. C. Agnis, M. D., - - - - - - - 25
Hypodermic, Injection of Medicine, by C. Hunter, - - - - - - 101
Near-Sightedness a Disease, - - - - - - - - - - - - 77
Opthalmic Disease, by R. B. Carter, F. R. C. S., - - - - - - - - 75

Phthisis and Uterine Disease, by J. H. Bennett, M. D., - - - - 75
Typhus Fever, by Dr. Gairdner, - - - - - - - - - - - - - 26
Tetanus, Pathology of, by J. L. Clark, F. R. S., - - - - - - - 99

### MISCELLANEOUS DEPARTMENT.

Aneurism, Case of, - - - - - - - - - - - - - - - 36
Atropia and Morphia, Antagonism of, by Drs. Mitchell, Kean and Morehouse, 78
American Medical Association, Delagates to, - - - - - - - - 291
Albumen in Urine, Detecting, - - - - - - - - - - - - 391
Amenorrhæa and Dysmenorrhœa Apiol, In Treating, - - - - - 394
American Medical Association, Annual Meeting, - - - - - - - 185, 442
Brinsmade Prize, - - - - - - - - - - - - - - - - - 291
Bromide of Potassium in Epilepsy, - - - - - - - - - - - 114
Bromide of Potassum, experimental investigation, - - - - - - - 245
Bifid Uterus and Double Vagina, - - - - - - - - - - - 351
Buffalo Medical and Surgical Journal, volume five, - - - - - - - 499
Blood, Transfusion of, - - - - - - - - - - - - - - - 484
Chloroform in Neuralgia and Muscular Rheumatism, - - - - - - - 396
Cholera Infantum, - - - - - - - - - - - - - - - - 352
Cholera Lectures, by Alonzo Clark, M. D., New York, - - - - - - 347
Cerebro-Spinal Meningitis without Spotted Fever, - - - - - - 27
Cholera—How to Mitigate it, - - - - - - - - - - - - - 153
Croup Treatment by Inhalation of Lime Water, - - - - - - - 155
Cow, Vaccine Matter from, - - - - - - - - - - - - - - 240
Chloroform in Convulsions, internal use, - - - - - - - - - 244
Cattle Disease, - - - - - - - - - - - - - - - - - 479
Diabetes, Diet in, by E. Smith, M. D., - - - - - - - - - - 79
Dysmenorrdœa associated with a peculiar form of the Cervi, by Robert Barnes, - - - - - - - - - - - - - - - - 104
Diphtheria, circular from Dr. Norton, - - - - - - - - - - 116
Death of Professor T. Childs, - - - - - - - - - - - - 117
Disinfecting and Deoderising, new method, - - - - - - - - 156
Dysmenorrhœa, Employment of Apiol in, - - - - - - - - - 394
Diseased Meat, - - - - - - - - - - - - - - - - - 346
Epilepsy treated by Promide of Potassium, - - - - - - - - - 114
Experimental Investigations on Promide of Potassium, - - - - - 245
Fifty-ninth Annual Meeting of the New York State Medical Society, - - 283
Fever, Typhoid, in Pigs, - - - - - - - - - - - - - - 149
Gun-shot Wounds, Treatment of, - - - - - - - - - - - - 282
Hick's U. S. Army Hospital, - - - - - - - - - - - - - 449
Herald of Health, - - - - - - - - - - - - - - - - 505
Insanity, Puerperal, - - - - - - - - - - - - - - - 29
Injury, Severe Case of, - - - - - - - - - - - - - - 31
Lime Water, Inhalation of, in Croup, - - - - - - - - - - 155
Labor, Mortality of Child-bed as affected by number, - - - - - 243
Lectures on Cholera, by Prof. A. Clark, - - - - - - - - - 347
Local Injections in Neuralgia, - - - - - - - - - - - - 398
Meningitis, Cerebro-Spinal, without Spotted Fever, - - - - - - 27
Menstruation, Treatment of Painful, - - - - - - - - - - 144
Merritt Cash Prize, - - - - - - - - - - - - - - - - 290
Mortality in New York city, by Cyrus Ramsy, M. D., - - - - - 475
Murrain in Palestine, - - - - - - - - - - - - - - - 464
Non-transmission of Syphilis by Vaccination, - - - - - - - - 117
New York Academy of Medicine on Cholera, - - - - - - - - 504
Paralysis of Hand and Fore-arm from Smoking, - - - - - - - 397
Puerperal Insanity, - - - - - - - - - - - - - - - - 29
Proper Diet in Diabetes, - - - - - - - - - - - - - - 79
Purulent Infection, - - - - - - - - - - - - - - - - 80
Purgatives, Habitual Use of, - - - - - - - - - - - - - 115
Syphilization, - - - - - - - - - - - - - - - - - - 109

Sterile Condition, Influence of Uterine Displacement, - - - - - 147
Smoking, Paralysis from, - - - - - - - - - - - - - - 397
Transmission of Syphilis by Vaccination, - - - - - - - - - - 117
Uterine Displacement and Sterility, - - - - - - - - - - - - 147
University of Pennsylvania, - - - - - - - - - - - - - 159
Vaccine Matter from the Cow, - - - - - - - - - - - - - 240
Ventilation of Hospitals, - - - - - - - - - - - - - - 241
Vagina, a Case of Double, - - - - - - - - - - - - - - 351
Will, Dr. Mott's, - - - - - - - - - - - - - - - - - 235

### EDITORIAL DEPARTMENT.

American Medical Association, - - - - - - - - - - - - 40, 307
American Medical Association, Transactions of, - - - - - - - 84, 124
Asiatic Cholera, - - - - - - - - - - - - - - - - - 160
Artificial Arms and Legs, - - - - - - - - - - - - - - 114
Atlantic Monthly, - - - - - - - - - - - - - - - - 420
American Educational Monthly, - - - - - - - - - - - - 309
Atlantic Medical and Surgical Journal, - - - - - - - - - - 458
Bellevue Hospital, case of Leprosy, - - - - - - - - - - - 34
Bee Sting, Death from, - - - - - - - - - - - - - - - 36
Bellevue Hospital Medical College, Lectures in, - - - - - - - - 124
Berkshire Medical College Commencement, - - - - - - - - - 169
Blatchford, Dr. T, W., Death of, - - - - - - - - - - - - 251
Buffalo Superior Court, - - - - - - - - - - - - - - - 356
Buffalo Medical College Commencement, - - - - - - - - - - 371
Braithwaite's Retrospect, Appendix to, - - - - - - - - - - 420
Braithwaite's Retrospect, - - - - - - - - - - - - - - 420
Biography, American Medical, - - - - - - - - - - - - - 459
Cholera, Reasons for fearing an Epidemic, - - - - - - - - - 118
Cholera, Communicability of, and Prevention, - - - - - - - - 298
Cholera Conference, - - - - - - - - - - - - - - - - 308
Court of Buffalo. - - - - - - - - - - - - - - - - - 356
Correction, - - - - - - - - - - - - - - - - - - - 460
Detroit Preparatory School of Medicine, - - - - - - - - - - 114
Dispensatory in Buffalo, - - - - - - - - - - - - - - - 254
Epidemic of Cholera, Reasons for Fearing, - - - - - - - - - 118
Expectant Medicine, Medical Prize, - - - - - - - - - - 169, 209
Erie County Medical Society, - - - - - - - - - - - - 254, 460
Ether, - - - - - - - - - - - - - - - - - - - - - 459
Flint, Jr., Austin, Physiology by, - - - - - - - - - - - - 36
Fœtus, Retention of Urine in, - - - - - - - - - - - - - 310
Gail Hamilton, - - - - - - - - - - - - - - - - - - 460
Human Milk, Substitute for, - - - - - - - - - - - - - - 380
Hamilton, Prof. Frank H., new edition of his work on Fractures and Dislocations, - - - - - - - - - - - - - - - - - - - 458
Hypodermic Injection, Danger of, - - - - - - - - - - - - 459
Intra-Uterine Variola, - - - - - - - - - - - - - - - 310
Journals, New, - - - - - - - - - - - - - - - - - - 252
Journals, New Medical, - - - - - - - - - - - 252, 306, 379, 458
Kiss, Syphilis communicated by, - - - - - - - - - - - - 460
Leprosy, Report of a case, - - - - - - - - - - - - - - 34
Lectures in Buffalo, - - - - - - - - - - - - - - - - 123
Lectures in Bellevue Hospital Medical College, - - - - - - - - 124
Legal Liabilities of Physicians and Surgeons, - - - - - - - - 353
Literary Journal, New, - - - - - - - - - - - - - - - 309
Miami Medical College, - - - - - - - - - - - - - - - 124
Medical and Surgical Monthly, - - - - - - - - - - - - - 168
Medical Record, - - - - - - - - - - - - - - - - - - 310
Ovarian Tumor, - - - - - - - - - - - - - - - - - - 84
Os Uteri, Division of, - - - - - - - - - - - - - - - - 40
Opium deoderized, Tinct., - - - - - - - - - - - - - - 506

Premium offered, - - - - - - - - - - - - - - - - 124
Physicians' visiting list, - - - - - - - - - - - - - - - 168
Personal, - - - - - - - - - - - - - - - - - - 253
Prevention and communicability of cholera, - - - - - - - - - 298
Pharmacy, Legitimate, - - - - - - - - - - - - - - 506
Pathological specimens, reception of, - - - - - - - - - - 379
Reasons for fearing cholera, - - - - - - - - - - - - - 118
Richmond Medical Journal, - - - - - - - - - - - - - 168
Retention of urine in the fœtus - - - - - - - - - - - - 315
Stimulants in disease, - - - - - - - - - - - - - - - 32
Sub-cutaneous injections, danger from, - - - - - - - - - - 170
Successful removal of uterus and both ovaries by abdominal section, - 254
Substitutes for human milk, - - - - - - - - - - - - - 308
Typhoid Fever in Western New York, its increase and prevalence, - - 81
Transactions of America Medical Association, - - - - - - 84, 124
University of Buffalo commencement, - - - - - - - - 301, 371
Variola intro uterine, - - - - - - - - - - - - - - - 310
Valedictory address by E. N. B. Smith, M. D., - - - - - - - 371

### BOOKS REVIEWED.

Annual address before the Medical Society of the county of Oneida, by C. B. Coventry, M. D., - - - - - - - - - - - - - - - 162
Contributions to practical surgery, by W. H Van Buren, - - - - - 39
Circular Mo. 6, - - - - - - - - - - - - - - - - - 374
Disease, injuries, and malformations of the rectum and anus, by T. J. Ashton, M. D., - - - - - - - - - - - - - - - - 305
Essentials of Materia Medica and Therapeutics, by Alfred Garrod, M. D., F. R. S. - - - - - - - - - - - - - - - - - 83
Hand-book of skin diseases, by Thomas Hillier. M. D., - - - - - 38
Lectures on Inflammation, by John H. Packard, M. D., - - - - - 161
Lectures on Diseases of the Stomach, by Wm. Brinton, F. R. S., - - 213
Laryngoscope, in diseases of the throat, by M. D. McKenzie, M. D., - - 304
Materia Medica, for the use of students, by J. B. Biddle, M. D., - - 250
Obscure diseases of the brain, by F. Winslow, M. D., D. C. L., - - - 454
Practice of Medicine and Surgery applied to the diseases and accidents incident to women, by, W. H. Byford, A. M., M. D., - - - - - 212
Pressure in the treatment of Gonorrhœa and Purulent Ophthalmia, by Joseph L. Hildreth, U. S. V., - - - - - - - - - - - - 249
Practice of Medicine, by Thomas H. Tanner, M. D., F. L. S., - - - 302
Principles of Surgery, by James Syme, F. R. S. E. - - - - - - 303
Principles and Practice of Medicine, by Austin Flint, M. D., - - - 413
Physiology of Man, by Austin Flint, Jr., M. D., - - - - - - 417
Renewal of Life, clinical lectures by Thomas King Chambers, M. D.,- - - 37
Researches on the medical properties of nitrous oxide, by Geo. J. Ziegler, M.D., 121
Rhinoscopy and Laryngoscopy, by Dr. F. Shmelder, - - - - - - 416
Sleep and Insomnia, by W. A. Hammond, M. D., - - - - - - 39
Student's book of Cutaneous Medicine and Disease of the Skin, by Erastus Wilson, F. R. S., - - - - - - - - - - - - - - 247
Stimulants and Narcotics—their mutual relation, &c., by F. B. Austin, M. D., M. R. C. P., - - - - - - - - - - - - - - - - 248
Specialties in Medicine, by Henry D. Noyes, M. D., - - - - - - 250
Transactions of the American Medical Association, volume XVI., - - 456
Transactions of the Medical Society of the State of New York, for 1866 - 502
Wakefulness, with lectures on the Philosophy of Sleep, by W. A. Hammond, M., D., - - - - - - - - - - - - - - - - - 378
West on Diseases of Women, - - - - - - - - - - - - - 457
Books received, - - - 40, 123, 169, 213, 215, 306, 378, 419, 458, 504

potassium administered in thirty grain doses was found to be beneficial.

The book is a re-print from the New York Medical Journal for May and June. To those interested in this variety of disease, this work will be found exceedingly valuable. K.

---

### Books and Pamphlets Received.

National Lyrics. By John Greenleaf Whittier, with illustrations by George G. White, H. Fenn, and Charles A. Barry. Boston: Ticknor & Fields, 1865.

Songs for all Seasons. By Alfred Tennyson, with illustrations by D. Maclise, T. Cheswick, S. Eytinge, C. A. Barry, H. Fenn, and G. Perkins. Boston: Ticknor & Fields, 1865.

